# Hospital-Based Epidemiological Study of Distal Radius Fractures at a Hand and Microsurgery Unit in Malaysia

**DOI:** 10.7759/cureus.79795

**Published:** 2025-02-27

**Authors:** Allan Ravi Rajagopal, Sheena Toyat, Mohd Firdaus Mohd Aslam, Syahril Rizal Arsad, Rashdeen Fazwi Muhammad Nawawi

**Affiliations:** 1 Hand and Microsurgery Unit, Department of Orthopedics, Hospital Selayang, Batu Caves, MYS

**Keywords:** demographic, distal radius, epidemiology, fracture, medical record review

## Abstract

Objective: Distal radius fractures (DRFs) are the most common upper limb fractures in clinical practice. Due to the absence of a formal national registry describing the characteristics of DRFs in Southeast Asia, this study aims to provide hospital-based epidemiological data to bridge this gap in the literature.

Methods: We conducted a retrospective study in the Hand and Microsurgery Unit of Hospital Selayang, Batu Caves, Malaysia, which included all patients who sustained non-pathological DRFs over 24 months, from 01 January 2022 to 31 December 2023. Medical records were analyzed in terms of detailed demographic data, fracture characteristics, and mode of treatment.

Results: Over the two-year study period, we identified 446 patients with DRF, totaling 450 DRFs (four patients had bilateral injuries). Male patients outnumbered female patients 114 (64%) to 63 (36%) in 2022 and 145 (54%) to 124 (46%) in 2023. The mean age of patients was significantly higher in 2023 compared to 2022 (54.51 ± 22.20 vs. 45.25 ± 23.64 years, respectively; p < 0.0001). The most common fracture type was type A, based on the Orthopaedic Trauma Association(OTA) classification, accounting for 83.6% of cases in 2022 and 87.4% of cases in 2023. In terms of treatment, most DRFs were treated conservatively as opposed to operatively (77.4% vs. 71.3%), with plate fixation being the most common operative intervention, accounting for 57.5% of cases in 2022 and 73.3% of cases in 2023.

Conclusion: The incidence of DRF was higher among male patients than among female patients in our study population, with an increasing annual trend. Most cases were the result of low-impact trauma and were closed fractures that were conservatively managed.

## Introduction

Distal radius fractures (DRFs), which occur within 3 cm from the articular surface between the radius and the proximal row of the carpal bones, are one of the most common fractures of the upper limbs [1]. The incidence of DRF exhibits a bimodal age distribution, mainly affecting younger individuals who experience high-impact trauma, such as motor vehicle accidents. However, elderly female patients who fall on an outstretched hand make up a significant cohort, most likely due to osteoporosis [2].

Most population-based epidemiological studies that investigate fracture characteristics and management profiles of DRFs involve data from developed neighboring countries such as Sweden, Denmark, and Japan [3-5]. However, in Southeast Asian countries such as ours, where nationwide/trauma registries are not available, our best alternative is to investigate data from tertiary hospitals specializing in hand and microsurgery, such as Hospital Selayang in Batu Caves, Malaysia.

In this study, our objective was to fill the above-mentioned gap in local literature by describing the demographic profile, fracture characteristics, and treatment modalities of DRF over a two-year duration from the Hand and Microsurgery Unit of Hospital Selayang in Batu Caves, Malaysia.

## Materials and methods

This was a retrospective study conducted in the major Hand and Microsurgery Unit of Hospital Selayang, Batu Caves, Malaysia. We identified all patients who sustained non-pathological DRFs for a study duration of 24 months, from 01 January 2022 to 31 December 2023. Distal radius fractures were defined as involving the distal 3 cm of the radius, including open fractures, bilateral fractures, and in association with other injuries. Hospital Selayang is a tertiary hospital with a subspecialized hand and microsurgery unit that receives all complex hand pathologies in the region.

Medical records of patients with non-pathological DRFs were identified at our center during the study period using the International Classification of Diseases, Tenth Revision, Clinical Modification (ICD-10-CM) as S52.50, S52.51, S52.60, and S52.61 [6]. The study was approved by the National Medical Research Register (NMRR ID-22-01245-PLR-IIR)., and the hospital’s institutional ethics committee waived the need for informed consent because of the retrospective, observational nature of the study.

Patient records were analyzed for detailed demographic characteristics and mode of treatment. Demographic information, including patient age, gender, and ethnicity, was documented. Clinical information was also recorded, including mechanism of injury, fracture type (open fracture or closed fracture), Orthopaedic Trauma Association (OTA) classification [7], involvement of ulnar styloid fracture, and treatment modality (conservative or operative). Conservative treatment was defined as the absence of a relevant surgical treatment within 21 days of diagnosis. Operative treatment was defined as a relevant surgical procedure performed within 21 days of diagnosis. Operative treatment was then divided into several surgical procedures, including external fixation, plate fixation, Kirschner wire (K-wire) fixation, and screw fixation.

Statistical analysis

All data were analyzed using IBM SPSS Statistics Software Version 25 (IBM Corp., Armonk, NY). Categorical variables are expressed as frequency and percentage, and continuous variables are presented as mean ± standard deviation (SD). Between-year comparisons were performed with the Student’s t-test. A p-value of <0.05 was considered statistically significant for all analyses.

## Results

A total of 446 patients were identified with 450 DRFs (four patients had bilateral injuries) over the study period of two years. There was a male preponderance throughout the study duration, with male-to-female ratios of 114 to 63 in 2022 and 145 to 124 in 2023. Distal radius fractures occurred at all ages in our study population, with the 20-59 years being the most affected age group (Table 1).

**Table 1:** Demographic and clinical data All numerical data are expressed as mean ± SD, and categorical data as n (%); p-values based on independent-samples t-test; *p-value < 0.05 indicates statistically significant differences

Variable	Year 2022 (N=177)	Year 2023 (N=269)	p-value
Number of overall fractures	181	269	0.384
Age (years)	45.25 ± 23.64	54.51 ± 22.20	< 0.0001*
Gender			0.3319
Male	114 (64%)	145 (54%)	
Female	63 (36%)	124 (46%)	
Age group (years)			0.341
0–19	29 (16)	5 (3)	
20–59	87 (49)	152 (56)	
≥60	61 (35)	112 (41)	
Ethnicity			0.4217
Malay	86 (49)	110 (41)	
Chinese	49 (28)	89 (33)	
Indian	30 (17)	49 (18)	
Other	12 (6)	21 (8)	

In terms of ethnicity, the majority of the study population was Malay for both years, representing 49% and 41% of patients in 2022 and 2023, respectively, followed by patients of Chinese, Indian, and other ethnicities for both years. These results mirrored the makeup of the local population, which is majority Malay. The mean age of patients was significantly higher in 2023 compared to 2022 (54.51 ± 22.20 vs. 45.25 ± 23.64 years, respectively; p < 0.0001) (Table 1).

The most common fracture mechanism was low-impact falls on outstretched hands, accounting for nearly 60% of cases, with 106 cases in 2022 and 154 cases in 2023. Closed fractures accounted for almost all cases, representing 97.2% of cases in 2022 and 91.6% of cases in 2023. According to OTA classification, fractures were grouped into type A (extra-articular), type B (partial articular), or type C (complete articular) [7]. Based on the interpretation of wrist radiographs, the most common fracture type for both years was type A (83.6% and 87.4%), followed by type B (10.2% and 7.2%) and type C (6.2% and 5.4%). We also noted that most DRFs did not involve ulnar styloid fractures for both years (73.4% and 70.1%). In terms of treatment, most DRFs were treated conservatively as opposed to operatively (77.4% vs. 71.3%). Among the different operative methods, plate fixation was the most common type for both 2022 and 2023 (57.5% and 73.3%) (Table 2).

**Table 2:** Comparison of fracture profiles between 2022 and 2023 All numerical data are expressed as mean ± SD, and categorical data as n (%); p-values based on independent-samples t-test; *p-value < 0.05 indicates a statistically significant difference

Variable	Frequency (n)/Percentage (%)		p-value
	2022 (N=177)	2023 (N=269)	
Mechanism of injury			0.6586
Fall onto an outstretched hand	106 (59.9)	154 (59.0)	
Motor vehicle accident	60 (33.9)	87 (33.3)	
Fall from height	9 (5.1)	16 (6.1)	
Crush injury	1 (0.6)	3 (1.1)	
Other	1 (0.6)	1 (0.4)	
Fracture type			0.7897
Closed fracture	172 (97.2)	239 (91.6)	
Open fracture	5 (2.8)	22 (8.4)	
Orthopaedic Trauma Association classification [7]			0.7569
Type A	148 (83.6)	228 (87.4)	
Type B	18 (10.2)	19 (7.3)	
Type C	11 (6.2)	14 (5.4)	
Involvement of ulnar styloid fracture			0.5973
Yes	47 (26.6)	78 (29.9)	
No	130 (73.4)	183 (70.1)	
Treatment modality			
Conservative	137 (77.4)	186 (71.3)	0.6733
Operative	40 (22.6)	75 (28.7)	0.4332
External fixation	5 (12.5)	8 (10.7)	
Plate fixation	23 (57.5)	55 (73.3)	
Kirschner wire (K-wire) fixation	10 (25.0)	9 (12.0)	
Screw fixation	2 (5.0)	3 (4.0)	

## Discussion

In this study, the overall number of DRFs in the Hand and Microsurgery Unit of Hospital Selayang was 181 in 2022 and 269 in 2023. In comparison, a population-based register study in Denmark reported an annual incidence of 228 per 100,000 population, with an increment of 31% over a study period of 20 years [8]. Similarly, a population-based study in Japan found an annual incidence of DRF of 158.0-272.6 per 100,000 population [5]. In comparison with these developed countries, which have established, nationwide DRF registries, developing countries such as Malaysia have a paucity of such data. As such, our hospital-based epidemiological study serves to fill this gap in the literature. When we look at similar Asian countries such as India, DRFs account for 3.1% of all emergency department visits referred to by orthopedics teams [9].

In contrast with most studies on the incidence of DRF, there was a preponderance of male patients among our study population for both years. For example, Candela et al. reported a higher prevalence of DRF in female patients, with a female-to-male ratio of 3:1 [10,11]. However, a US-based study utilizing data from the National Trauma Data Bank (2016-2019) reported a similar male preponderance of 65.1% [4]. Furthermore, another study done in rural southern Norway reported a male preponderance of DRF, with younger age, male gender, summer seasons, and living in rural areas being independent risk factors for increased risk of high-energy DRF [12].

We could not identify a bimodal distribution of DRF prevalence in young adults or elderly female patients, as cited in most other studies [13]. However, we did identify mean ages of 45.25 ± 23.64 years and 54.51 ± 22.20 years for 2022 and 2023, respectively.

In terms of injury characteristics, falling on outstretched hands was the most common mechanism of trauma resulting in DRF for both years, followed by motor vehicle accidents. These low-impact fractures were also mostly closed fractures, similar to data from Swedish and Danish DRF fracture registries [3]. In terms of OTA classification [7], the most common fracture type was type A, as seen in a study conducted in Vigo, Spain [14].

In terms of treatment modality, most DRFs were managed conservatively. This was in contrast with most registry data from developed countries, which have consistently shown increased rates of surgical treatment from 9% to 16% to 13% to 23% [15]. There has been intense discussion as to whether surgical or conservative treatment is more effective for treating DRFs. Based on radiographic outcomes, previous systematic reviews have shown that surgical interventions are more effective than conservative management. However, in terms of functional outcomes, there are no clinically significant differences between conservative treatment and various types of surgical interventions available for DRFs [15].

We have found that differences in management options (i.e., being more inclined to manage DRFs conservatively) may be influenced by socioeconomic factors [16]. There are vast differences in healthcare systems between the advanced and emerging economies in Southeast Asia. For example, in our region, Singapore possesses an advanced economy that is similar to developed nations, whereas, in Malaysia, the healthcare system is burdened by a low per-capita income, overcrowding, inadequate resources, and even socio-cultural factors [17].

Study limitations

In our study, unlike most population-based registry studies in developed regions, we were not able to calculate the incidence rate of DRFs among our local population. In addition, we did not study the functional outcomes of both conservatively and surgically managed DRFs in our institution, which would give us a better perspective on whether we should be more inclined to conservatively manage most DRFs, as we currently do.

## Conclusions

There is limited data concerning the epidemiology of DRF from developing Asian countries. The incidence of DRFs at Hospital Selayang was higher among male patients than among female patients, with an increasing yearly trend and mostly affecting those between 20 and 59 years of age. Most cases were caused by low-impact trauma and were closed fractures that were conservatively managed. These differences in presentation and management may be explained by barriers to healthcare services, cost factors, and the socio-cultural data that is worthwhile to investigate if it influences the choice of treatment.

## References

[1] Gutiérrez-Espinoza H, Araya-Quintanilla F, Cuyul-Vásquez I, Gutiérrez-Monclus R, Reina-Gutiérrez S, Cavero-Redondo I, Arenas-Arroyo SN (2023). Effectiveness and safety of different treatment modalities for patients older than 60 years with distal radius fracture: a network meta-analysis of clinical trials. Int J Environ Res Public Health.

[2] de Alencar Neto JB, Jales CD, Coelho JV, de Souza CJ, Cavalcante ML (2022). Epidemiology, classification, and treatment of bilateral fractures of the distal radius. Acta Ortop Bras.

[3] Rundgren J, Bojan A, Mellstrand Navarro C, Enocson A (2020). Epidemiology, classification, treatment and mortality of distal radius fractures in adults: an observational study of 23,394 fractures from the national Swedish fracture register. BMC Musculoskelet Disord.

[4] Chinta SR, Cassidy MF, Tran DL, Brydges HT, Ceradini DJ, Bass JL, Agrawal NA (2024). Epidemiology of distal radius fractures: elucidating mechanisms, comorbidities, and fracture classification using the national trauma data bank. Injury.

[5] Ando J, Takahashi T, Ae R (2023). Epidemiology of distal radius fracture: a regional population-based study in Japan. BMC Musculoskelet Disord.

[6] (2015). ICD-10-CM. https://www.cdc.gov/nchs/icd/icd-10-cm/index.html.

[7] Marsh JL, Slongo TF, Agel J (2007). Fracture and dislocation classification compendium - 2007: Orthopaedic Trauma Association classification, database and outcomes committee. J Orthop Trauma.

[8] Viberg B, Tofte S, Rønnegaard AB, Jensen SS, Karimi D, Gundtoft PH (2023). Changes in the incidence and treatment of distal radius fractures in adults - a 22-year nationwide register study of 276,145 fractures. Injury.

[9] Anil A, Acharya AM, Bhat AK (2022). A six-year clinical profile of distal radius fractures in a South Asian population. J Hand Surg Asian Pac Vol.

[10] Candela V, Di Lucia P, Carnevali C, Milanese A, Spagnoli A, Villani C, Gumina S (2022). Epidemiology of distal radius fractures: a detailed survey on a large sample of patients in a suburban area. J Orthop Traumatol.

[11] Soerensen S, Larsen P, Korup LR (2024). Epidemiology of distal forearm fracture: a population-based study of 5426 fractures. Hand (N Y).

[12] Diamantopoulos AP, Rohde G, Johnsrud I, Skoie IM, Hochberg M, Haugeberg G (2012). The epidemiology of low- and high-energy distal radius fracture in middle-aged and elderly men and women in Southern Norway. PLoS One.

[13] Azad A, Kang HP, Alluri RK, Vakhshori V, Kay HF, Ghiassi A (2019). Epidemiological and treatment trends of distal radius fractures across multiple age groups. J Wrist Surg.

[14] Zugasti-Marquínez J, García-Reza A, Domínguez-Prado DM (2022). Epidemiological study of distal radius fractures in the sanitary area of Vigo (Article in Spanish). Revista Espanola de Cirugia Ortopedica Traumatologica.

[15] Gutiérrez-Espinoza H, Araya-Quintanilla F, Olguín-Huerta C, Gutiérrez-Monclus R, Valenzuela-Fuenzalida J, Román-Veas J, Campos-Jara C (2022). Effectiveness of surgical versus conservative treatment of distal radius fractures in elderly patients: a systematic review and meta-analysis. Orthop Traumatol Surg Res.

[16] Koo OT, Tan DM, Chong AK (2013). Distal radius fractures: an epidemiological review. Orthop Surg.

[17] Sebastin SJ, Chung KC (2012). An Asian perspective on the management of distal radius fractures. Hand Clin.

